# QbD Consideration for Developing a Double-Layered Tablet into a Single-Layered Tablet with Telmisartan and Amlodipine

**DOI:** 10.3390/pharmaceutics14020377

**Published:** 2022-02-08

**Authors:** Joo-Eun Kim, Young-Joon Park

**Affiliations:** 1 Department of Pharmaceutical Engineering, Catholic University of Daegu, Hayang-Ro 13-13, Hayang-Eup, Gyeongsan City 38430, Gyeongbuk, Korea; 77jooeun@naver.com; 2 College of Pharmacy, Ajou University, Worldcup-ro 206, Yeongtong-gu, Suwon-si 16499, Korea

**Keywords:** telmisartan, amlodipine, quality-by-design, design of experiment, risk assessment, design space, bioequivalence

## Abstract

The aim of this study was to develop a single-layered version of commercially available Twynstar^®^ (Telmisartan + Amlodipine) double-layered tablets to improve the dosing convenience. A quality-by-design approach was applied to develop the single-layered version. To evaluate the range and cause of risks for a single-layered tablet in the formulation design research, we used the tools of the risk assessment, initial risk assessment of preliminary hazard analysis and main risk assessment of failure mode and effect analysis to determine the parameters affecting formulation, drug dissolution, and impurities. The critical material attributes were the stabilizer and disintegrant, and the critical process parameters were the wet granulation and tableting process. The optimal range of the design space was determined using the central composite design in the wet granulation and tablet compression processes. The stabilizer, kneading time, and disintegrant of the wet granulation were identified as X values affecting Y values. The compression force and turret speed in the tablet compression were identified as X values affecting Y values. After deciding on the design space with the deduced Y values, the single-layered tablets were formulated, and their dissolution patterns were compared with that of the double-layered tablet. The selected quality-by-design (QbD) approach single-layered tablet formulated using design space were found to be bioequivalent to the Twynstar^®^ double-layered tablets. Hence, the development of single-layered tablets with two API using the QbD approach could improve the medication compliance of patients and could be used as a platform to overcome time-consuming and excessive costs and the technical and commercial limitations related to various multi-layered tablets.

## 1. Introduction

The telmisartan-amlodipine complex double-layered tablet is marketed as a commercial product under the trade name of Twynstar^®^ and is used to treat essential hypertension [1]. Twynstar^®^ is composed of amlodipine besylate and telmisartan freebase. Amlodipine besylate is benzenesulfonic acid: 3-O-ethyl 5-O-methyl 2-(2-aminoethoxymethyl)-4-(2-chlorophenyl)-6-methyl-1,4-dihydropyridine-3,5-dicarboxylate. It is a calcium channel blocker used to treat coronary artery disease and high blood pressure. Amlodipine relaxes blood vessels and improves blood flow. Amlodipine may be used if other medications are not adequate for heart-related chest pain or high blood pressure. In addition, amlodipine is a very useful calcium channel blocker, which shows pharmacological activity over a long period of time with a half-life of 30 to 50 h [2].

Telmisartan, 4-((2-*n*-propyl-4-methyl-6-(1-methylbenzimidazole-2-yl)-benzimidazole-1yl)methyl)-biphenyl-2-carboxylic acid, reduces blood pressure as a selective angiotensin II type 1 receptor blocker [3]. Telmisartan keeps blood vessels from narrowing, which improves blood flow and lowers blood pressure. Lowering blood pressure may lower the risk of a stroke or heart attack. The effectiveness of the combination therapy of telmisartan and amlodipine has already been proven through various studies. Specifically, in patients with severe hypertension with blood pressure of 180/95 mmHg or more; the fixed-dose combination therapy of two drugs and each monotherapy were compared, and the combined group showed a significant reduction in mean systolic blood pressure (up to 50 mmHg) [4]. In addition, the combination therapy of telmisartan and amlodipine showed a superior systolic blood pressure-lowering effect compared with each single drug after 1 week of treatment and showed sustained efficacy. In addition, another effect is reducing side effects such as reducing peripheral edema produced when amlodipine alone is used. By combining different antihypertensive drugs in this way, it is possible to more effectively treat hypertension through drug synergistic effects than through a single component administered by simultaneously targeting different targets that cause blood pressure increase through differentiated mechanisms. As the synergistic effect of these two drugs is revealed, the demand for a combination drug is gradually expanding in the hypertension drug market, and a combination drug including telmisartan and amlodipine is currently being released [5]. A prime example is Twynstar^®^. Twynstar^®^ is a commercially successful pharmaceutical product launched in 2010 by Boehringer Ingelheim. Since telmisartan is characterized by very low solubility in the physiological pH range of the gastrointestinal tract, an alkalinizing agent must be used to improve the solubility of telmisartan [6]. However, given that the alkalinizing agent has strong deliquescent properties, the telmisartan formulation absorbs moisture in the air by itself and dissolves within a few hours of being opened, causing great difficulty in terms of storage stability [7]. Moreover, due to the alkalinizing agent, the chemical structure of the drug compounded with telmisartan may be changed and the related substances may be released, which may have a fatal effect on the stability of the formulation. Therefore, as a way to solve this problem, Twynstar^®^ is manufactured as a double-layer tablet [8]. However, these may be inconvenient for elderly patients to consume due to the increased size of the tablet, and there also the disadvantages of low productivity and increased production cost because it requires special equipment such as a two-layer tablet press machine [9]. In particular, in the case of treatments for chronic diseases such as high blood pressure, it is essential to improve compliance because they often have a large reliance on drugs. In addition, it is challenging to maintain uniform quality due to difficulty of maintaining content uniformity in each layer of the tablet, poor mixing, and low quality of the mixture [10]. Therefore, our research team aimed to develop a single-layered version of a combination formulation that has the same efficacy as the conventional Twynstar^®^ and reduces the size of the tablet by half.

We explored methods that could reduce the tablet size by half, while maintaining the same efficacy and bioequivalence and that could solve the problems relating to quality, impurities, and mixing without the use of a double-layer tablet press machine. In the preliminary studies, we examined a method that could manufacture a smaller two-layered tablet by reducing the amount of excipients by half. However, when we reduced the size of the two-layered tablets, there was change in the dissolution pattern and bioequivalence. Therefore, our research team applied the quality-by-design (QbD) approach [11] to set the direction in which the elution pattern and related substances were not damaged in the single-layered tablets, there was no difference in bioequivalence, and the pK was also minimally affected [10].

To develop a bioequivalent formulation to turn the double-layered tablet into a single-layered tablet with telmisartan and amlodipine, we have applied QbD approach methods, which consist of building high quality into drug products by statistical design [12]. The QbD [13] approach methods ensure that the pharmaceutical development process is concentrated on providing a scientific understanding of how material and process parameters affect drug product performance. This knowledge enables the establishment of a design space, process parameter, process conditions, and drug product, as well as reasonable manufacturing controls that ensure the consistent accomplishment of all necessary quality targets, formulation design, and drug product requirements [14].

We can design the formulation and process by developing them based on the multivariate analysis of historical data, designed experiments, or both, to identify and characterize the critical to quality process parameters as well as the basic causes of variability [15]. Design of experiments (DoE) is used as a tool for identifying and optimizing critical material attributes (CMAs)-critical process parameters (CPPs) [16]. To optimize the formulation and process, statistical DoEs [17] are applied during the pharmaceutical process development and product development [18].

To increase the patient’s compliance by reducing the size of the tablet by half and to overcome various problems that occur during when manufacturing a single-layered tablet that has been developed from a double-layered tablet, our research team developed a single-layered tablet by applying a risk assessment (RA) using the QbD approach [19].

This novel single-layered tablet, manufactured using the QbD approach, was significantly smaller than the marketed tablets. This may be advantageous in increasing patients’ compliance, especially in various patients with hypertension who have difficulty swallowing. Furthermore, the efficacy and bioequivalence of the single-layered tablet were comparable with that of the commercial Twynstar^®^ tablet, and the problems relating to quality, impurities, and mixing could be addressed even without using a double-layer tablet press machine. Therefore, the results of this study provide theoretical evidence that when telmisartan and amlodipine are prepared as single-layered tablets, they have the same efficacy as conventional double-layer tablets, while also increasing patient compliance. Moreover, this introduction of single-layered tablets is expected to be effectively applied to elderly patients suffering from diseases such as hypertension.

## 2. Materials and Methods

### 2.1. Materials

Telmisartan potassium was purchased from Hwail-Pharmaceutical Co., Ltd. (Seoul, Korea). Amlodipine besylate was provided by Dr. Reddy’s Laboratories Ltd. (Mumbai, India). Telmisartan, amlodipine besylate, and related reference substances were purchased from a USP reference standards store of the United States Pharmacopeial Convention (Rockville, MD, USA). D-mannitol 200SD was provided by Merck & Co., Inc. (Kenilworth, NJ, USA). Potassium chloride was provided by Samchun Chemical Co., Ltd. (Pyeongtaek-si, Korea). Magnesium oxide (MgO) was provided by Tomita Pharmaceutical Co., Ltd. (Tokushima, Japan). Prosolv EASYTab and microcrystalline cellulose PH101 and croscarmellose sodium VIVASOL were purchased from JRS Pharma Co., Ltd. (Patterson, NY, USA). Crospovidone CL-F was provided by BASF Pharma Co., Ltd., (Rotherstraße, Berlin, Germany). Magnesium stearate was purchased from Merck KGaA (Frankfurter StraBe, Darmstadt, Germany). Colloidal silicon dioxide was provided by Evonic Health Care Co., Ltd., (Rellinghauser Straße, Essen, Germany). All other chemicals were of reagent grade and purchased commercially.

### 2.2. Initial Risk Assessment by Preliminary Hazard Analysis

The risk assessment (RA) was performed to select and evaluate the critical material attributes (CMAs) and critical process parameters (CPPs) that affect the critical quality attributes (CQAs). In this study, preliminary hazard analysis (PHA) was used as the initial RA tool [20]. The initial risk assessment was based on prior knowledge, screening experiments, and experience and information about pharmaceutical dosage form, obtained from published literature. In the PHA, the material attribute (MA) and process parameter (PP), obtained through preliminary studies, were listed [10,19]. CMA has a critical effect on CQA among various material attributes. CPP has a critical effect on the CQAs among various process parameters. For the final product, appearance, assay, content uniformity, impurities, and drug dissolution were selected as CQAs. Telmisartan potassium, amlodipine besylate, stabilizer, diluent, binding solution, disintegrant, glidant, and lubricant were selected as candidate MAs that affect CQAs. Screening, blending, granulation, drying, post-mixing and lubrication, tablet compression, and film coating were selected as candidate PPs that affect CQAs. Though PHA, a table is composed, in which the risk levels of various items are indicated by a color: green (low-risk), yellow (medium-risk), and red (high-risk), respectively. Green refers to a widely acceptable risk and no further studies such as DoE are needed, and yellow refers to a reasonably acceptable risk range, which may require the justification of further studies to reduce the risk. Red refers to an unacceptable risk that requires further studies to reduce the risk. This step indicates that action (such as DoE) should be taken, if necessary.

### 2.3. Main Risk Assessment by Failure Mode and Effect Analysis

**Figure 1:**
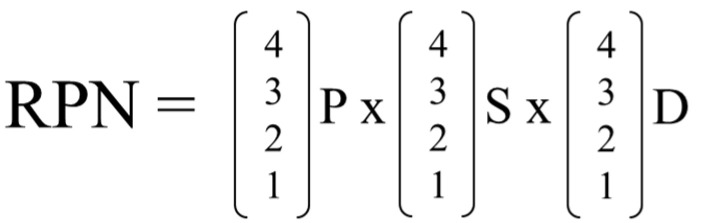
The calculation method of a risk priority numbers (RPN).

Quantitative failure mode and effect analysis (FMEA) was used for further accuracy and precision analysis of CMAs and CPPs [10]. The risk factors in the PHA were classified into wide categories, and the FMEA confirmed the failure modes that have the highest chance of causing drug deterioration of product quality, which means that each of the factors in the PHA will be ranked later in the FMEA analysis. The FMEA method can be used to perform the quantitative risk assessment, identifying the CQAs that have the most likely chance of causing deterioration of product quality. The FMEA was divided into two tables, one for CMAs and one for CPPs. Among the various MAs in the table of CMAs, the functional groups of APIs and excipients that are believerd to affect CQAs are listed in the FMEA of Table 1. Among the various PPs in the table of CPPs, the unit process of tablets that are considered to affect CQAs are listed in the FMEA of Table 1. As shown in Figure 1, the results of an FMEA are risk priority numbers (RPN) for each combination of failure mode severity, probability, and likelihood of detection [10]. The RPN defined as the overall failure risk, was evaluated based on three criteria: frequency of probability (P), effect severity (S), and detect ability (D) each of them rated on a scale from 1, as the low level of the mentioned criteria to 4, as the high level of the criteria [19].

The final values (RPN) were confirmed by the multiplication of the values registered for each of the three items [20]. CPPs and CMAs that rated as the highest risk levels were studied in detail in DoE. The value of failure modes with RPNs ≥40 were set as criteria for considering a failure requiring action such as DoE.

### 2.4. Design of Experiments

Before the development of a suitable experimental design which would allow an in-depth study of the formulation and process, some preliminary experiments were performed so as to establish a stable and reliable formulation process. These experiments aimed to define the optimal formulation process and formulation materials [21,22,23].

The Minitab^®^ program (version 19; Minitab Inc., State College, PA, USA) was used to perform the DoE. CMAs and CPPs through the PHA/FMEA of RA were selected as input (X) values. Then, in order to optimize a factor affected by the X values, the output (Y) value was adopted as the most important variable. Once the design of experiments was developed through CMAs and CPPs, analysis of variance (ANOVA) analysis was performed. Response surface design analysis using regression equation and ANOVA was optimized by selecting only the significant factors and this is for the optimized modeling of the DoE. Next, we determined the selected effect-factor through the pareto chart of standardized effect and residual plot. Finally, we confirmed whether the model was adequate through a lack of fit test. The *p* values were >0.05 and, hence, we could not refuse the null hypothesis for lack of conformity [10].

We evaluated the main, interaction, and quadratic effects of factors related to the properties of monolayered TA tablets and investigated the effect of each independent variable on the dependent variable. Variables with *p*-values less than 0.05 were regarded as statistically significant [19].

### 2.5. Development of the Design Space

The design, analysis, and plotting of contour plots and the various three-dimensional (3D) tasks were performed using Minitab^®^ software. Once the models were developed and validated, the design space was finally optimized through a multidimensional combination of all of the individual acceptance regions for CMA and CPP [24].

The effect of CMA and CPP was analyzed by factorial plots (main effect and interaction effects), contour plots, and response surface plots in each unit process. The design space represents the optimum design values of the actual operating space by superimposing the response contour plots.

After overlapping the contour maps of each response, response surface methods were used to identify the ideal window of the operability design space according to the acceptable range and failure edges with relation to the individual goals [25].

### 2.6. Preparation of Telmisartan Potassium and Amlodipine Besylate Tablets

The monolayered telmisartan potassium and amlodipine besylate (TA) tablets were manufactured according to the formulation compositions shown in the range of design space and preliminary studies, using the manufacture process as an order of (1) wet granulation method with a high shear mixer, (2) drying process with a fluid bed dryer, (3) blending with an intermediate bulk container, (4) lubrication, (5) tablet press, and (6) film coating.

Each monolayered TA tablet comprised 91.1 mg of telmisartan potassium and 6.94 mg of amlodipine besylate as the active pharmaceutical ingredients. We used telmisartan potassium, equivalent to 80 mg of telmisartan free-base, and amlodipine besylate, equivalent to 5 mg of amlodipine free-base in our formulation.

In addition, each tablet comprised D-mannitol, magnesium oxide (MgO), potassium chloride, microcrystalline cellulose (MCC), crospovidone, modified microcrystalline cellulose (prosolv SMCC), colloidal silicon dioxide, croscarmellose sodium, and magnesium stearate as the inactive pharmaceutical ingredients.

Using a laboratory mixer, telmisartan potassium and colloidal silicon dioxide were mixed for 3 min at 15 rpm. The obtained mixture, D-mannitol, crospovidone, MgO, and part of the MCC were further mixed at 15 rpm for 8 min using a laboratory mixer, and then the acquired mixture was sieved with a 20-mesh screen. For the wet granulation process, 99.5% anhydrous ethanol was added to this mixture to yield wet granules, impeller and chopper speed were maintained at 150 rpm and 2000 rpm, respectively, during the wet granulation process, using a laboratory high shear mixer for 5 min. Then, the granules were put in a fluid bed dryer (Glatt GPCG1, Ramsey, NJ, USA) and dried at 55 °C for 1.2 h so that the water content was 2.0% or less. The dried granules were sieved using a 0.065-inch screen, processed through a Cone Mill instrument (Hosokawa micron Ltd., Cheshire, UK), and the final weight was confirmed. Amlodipine besylate and part of the colloidal silicon dioxide were mixed for 3 min at 15 rpm using a laboratory mixer.

The milled granules and mixed amlodipine besylate were mixed with potassium chloride, croscarmellose sodium, part of the colloidal silicon dioxide, part of the modified MCC for 3 min and then finally mixed with magnesium stearate. Monolayered TA tablets were manufactured by compression using a rotary tablet press machine and coated with a film coating machine to an aimed hardness and mass weight of 11 Kp and 310 mg, respectively, as indicated in the design space [26].

### 2.7. Evaluation of Single-Layered TA Tablets

#### 2.7.1. Hardness Test

The hardness level of the single-layered TA tablets was assessed using the Model 8M bench top hardness tester (Pharmatron, Aesch, Switzerland). The singe-layered TA tablet was placed between the movable part of the instrument and the edges of the fixed part, and the hardness was measured in kiloponds.

#### 2.7.2. Friability

The friability of the monolayered TA tablets was measured using a FT 2 friability tester (Pharmatron model FT 2 friability tester, Aesch, Switzerland). Friability represents a measure of tablet strength, and this was calculated as a percentage using the below formula:

Friability (%) = [(w_1_ − w_2_)/w_1_] × 100, (1)

w_1_ is the weight of TA tablets before testing, and w_2_ is the weight of TA tablets after testing.

#### 2.7.3. Disintegration Test

The disintegration rate of the monolayered TA tablets was analyzed using a tablet disintegration tester (Pharmatron model DisiTest 20 tablet disintegration tester, Aesch, Switzerland). The disintegration test was performed according to the US Pharmacopeia (USP) by placing six monolayered TA tablets into a tablet disintegration tester.

#### 2.7.4. In Vitro Dissolution of TA Tablets

The dissolution test [27] was carried out according to the ‘telmisartan and amlodipine tablets’ dissolution test of U.S. Pharmacopeia (USP), with 900 mL of pH 7.5 ± 0.1 phosphate buffer (6.8 g of monobasic potassium phosphate dissolved in 1 L of water) as the dissolution medium at 37 ± 0.5 °C for telmisartan and 500 mL of 0.01 N hydrochloric acid for amlodipine besylate. The speed of the paddle was set to 75 rpm. The Twynstar^®^ and TA tablets, at an equivalent dose of 80 mg telmisartan base and 5 mg amlodipine besylate, were put into the dissolution tester (Agilent dissolution tester, Santa Clara, CA, USA). After 5, 10, 15, and 30 min, 4 mL of the dissolution medium was sampled and filtered using a membrane filter (0.45 μm). The concentration of telmisartan and amlodipine in the filtrated samples was analyzed using HPLC (Agilent Technologies, Santa Clara, CA, USA) at a UV detection wavelength of 237 nm. A Supelco Discovery C18 column (4.6 × 150 mm, 5 μm) (Sigma-Aldrich, Saint Louis, MO, USA) was used. The mobile phase was a 40:60 volume mixture of acetonitrile and aqueous buffer (pH 2.4, dissolve 2.72 g monobasic potassium phosphate in 1 L of water and pH adjusted with phosphoric acid). The flow rate was set to 1.0 mL/min, and the injection volume was set to 10 μL. The cumulative percentage of the released drug was calculated as the mean value of at least six tablets [10].

The in vitro dissolution profiles of the monolayered TA tablets and reference drug (Twynstar^®^) were compared using the similarity factor (f_2_), recommended by the Guidance for Industry of US Food and Drug Administration (FDA) [19].

#### 2.7.5. High-Performance Liquid Chromatography (HPLC) Analysis

The content and impurity level of telmisartan and amlodipine were analyzed using a high-performance liquid chromatography (HPLC) system [27] equipped with a UV/Vis detector (Agilent 1200 series, Santa Clara, CA, USA) and a separation module, according to the ‘telmisartan and amlodipine tablets’ of U.S. Pharmacopeia (USP). The assay of telmisartan and amlodipine content was performed using a Luna C18 column (4.6 × 250 mm, 5 μm; Phenomenex Inc., Torrance, CA, USA) as a stationary phase. The mobile phase, composed of a 40:60 volume mixture of acetonitrile and aqueous buffer (pH 6.0, 22 mM monobasic sodium phosphate dihydrate and 2 mL of trimethylamine dissolved in 1 L of water and pH adjusted with phosphoric acid), was filtered using a 0.22 μm membrane filter and then degassed using an online degasser. The column temperature, eluent flow rate, injection volume, and detection wavelength were 30 °C, 1.0 mL/min, 20 µL, and 257 nm, respectively. Calibration samples were prepared by accurately weighing and dissolving 40 mg of telmisartan and amlodipine each to obtain a stock solution, which was thereafter diluted to concentrations of 40.0–400 μg/mL and 5.0–100 μg/mL, respectively. The calibration curve was rectilinear with a correlation coefficient (r^2^) of 0.999, and the standard deviation (SD) of the accuracy and precision was less than 2% [28].

To analyze the number of impurities in telmisartan and amlodipine, a Zorbax SB C_18_ column (4.6 × 50 mm, 3.5 μm; Agilent Inc.) was used as the stationary phase. Mobile phases A and B were composed of acetonitrile: 23 mM ammonium acetate (pH 5.5 ± 0.1, adjusted with phosphoric acid) in the ratios of 20:80 and 65:35 (*v*/*v*), respectively. The following gradient program was followed: 0–5% B (0–5 min), 5–30% B (5–15 min), 30–55% B (15–35 min), 55–95% B (35–50 min), 95–100% B (50–65 min), 100% B (65–70 min), 100–5% B (70–75 min), and 5% B (75–80 min). The column temperature, eluent flow rate, injection volume, and detection wavelength were 30 °C, 1.0 mL/min, 20 µL, and 257 nm, respectively.

### 2.8. Pharmacokinetics

Ten *Canis familiaris* (beagle dogs) were randomly divided into two groups, and the reference drug (Twynstar^®^) and the test drug (monolayered TA tablets) were administered orally under fasting conditions, using a two-cycle single-dose crossover design. Between each dosing, there was a 1-week drug washout period. After oral drug dosing, blood samples (3.0 mL) were collected from the jugular vein at 0, 0.25, 0.5, 1, 2, 4, 6, 8, 12, 24, 36, 48, and 72 h [29]. The blood samples were immediately centrifuged for 10 min at 3000 rpm, and the plasma was stored at −70 °C until the analysis. The plasma concentrations of telmisartan and amlodipine in beagle dogs administering the reference drug (Twynstar^®^) or the monolayered TA tablets were analyzed through liquid chromatography-tandem mass spectrometry (LC-MS/MS). The Waters^®^ Micromass^®^ Quattro Premier XE Tandem quadrupole mass spectrometer (Quattro Premier XE, Waters, Milford, MA, USA), interfaced with an ultraperformance LC (UPLC, Alliance HT 2795, Waters, Milford, MA, USA), and equipped with a Unison UK-C_18_ column (75 × 2.0 mm internal diameter, 3 μm, Imtakt, Ringoes, NJ, USA) for telmisartan and a Luna Phenyl-Hexyl (100 × 2.0 mm, 3 μm, Phenomenex Inc., Torrance, CA, USA) for amlodipine, were used.

The stock solution of telmisartan was prepared by adjusting the concentration of 200 μg/mL in methyl alcohol, which was thereafter diluted with the blank serum to a concentration range of 10, 30, 50, 100, 500, 1000, 5000, and 20,000 ng/mL as the plasma standard. To prepare the test solution and standard solution, 10 μL of an internal standard solution (50 μg/mL of telmisartan-d7 in 50% methyl alcohol) was added to 50 μL of both standard plasma and test plasma. After adding 1000 μL of acetonitrile, centrifuging for 10 min at 3000 rpm and vortexing for 1 min, 2 μL of supernatant was analyzed by LC-MS/MS [10]. A 2 μL sample was separated chromatographically through isocratic conditions for the mobile phase (A:B, 35:65, *v*/*v*). 0.1% formic acid in 10 mM ammonium formate in distilled water was used as mobile phase A and 99.99% acetonitrile as B. The flow rate was set to 0.35 mL/min. The calibration curve was drawn with the telmisartan peak area ratio to the peak area ratio of the internal standard (of telmisartan-*d*_7_) obtained. We also performed assay validation with linearity, specificity, accuracy, and precision. The calibration curve was rectilinear with a correlation coefficient of 0.99901 to 0.99993, and the standard deviation (SD) of the accuracy and precision was less than 2%.

The stock solution of amlodipine was prepared by adjusting the concentration to 1000 μg/mL in methyl alcohol, which was thereafter diluted with the blank serum to a concentration range of 1, 2, 5, 10, 50, 100, and 200 ng/mL as the plasma standard.

To prepare the standard solution and the test solution, 100 μL of the internal standard solution (100 ng/mL of amlodipine-d4 in 50% methyl alcohol) was added to 10 μL of both standard plasma and test plasma. After adding 3 mL of methyl tert-Butyl ether, centrifuging for 5 min at 3000 rpm and vortexing for 3 min, 7 μL of supernatant was analyzed by LC-MS/MS. A 7 μL sample was separated chromatographically using isocratic conditions for the mobile phase (A:B, 20:80, *v*/*v*). An amount of 5 mM of ammonium formate in distilled water was used as mobile phase A and 99.99% acetonitrile was used as B. The flow rate was set to 0.32 mL/min. The calibration curve was drawn, comparing the obtained amlodipine peak area ratio with the obtained peak area ratio of the internal standard (of amlodipine-*d_4_*). We also performed assay validation with linearity, specificity, accuracy, and precision. The calibration curve was rectilinear with a correlation coefficient of 0.99936 to 0.99976, and the SD of the accuracy and precision was less than 2%.

### 2.9. Statistical Analysis

The data are expressed as mean ± SD. Comparison of the mean between groups was performed through single-factor variance analysis, and pairwise comparisons were performed using the least significant difference test. For *p* values of 0.05 or less, the difference was regarded as statistically significant. All statistical analyses were performed through Minitab^®^ software (Version 18, Minitab Inc., University Park, PA, USA). Pharmacokinetic parameters, including total area under the plasma concentration-time curve from time zero to infinity (AUC_0–∞_), time to reach C_max_ (T_max_), maximum plasma drug concentration (C_max_), and half-life (t_1/2_) were calculated using the WinNonlin^®^ software (Pharsight Co., Cary, NC, USA) from Crystal Genomics Pharmaceutical Co., Ltd. (Seongam, South Korea).

## 3. Results and Discussion

### 3.1. Initial Risk Assessment of the Critical Material Attributes and Critical Process Parameters Affecting Critical Quality Attributes

In the preliminary studies, the approximate monolayered formulation was designed taking into account the compatibility and stability between the APIs and inactive ingredients. Amlodipine besylate salt exhibits high decomposition in a basic medium, but the alkalization agent used in our study was an insoluble alkalizer, not a soluble alkalizer with strong deliquescent properties. In addition, the two active pharmaceutical ingredients used in this study of monolayered tablets were telmisartan potassium salt and amlodipine besylate salt. Telmisartan potassium is a non-hygroscopic substance.

Original telmisartan formulations have a process of Na+ salt adhesion to telmisartan to improve solubility. In order to attach sodium salt to the telmisartan freebase, substances with strong deliquescent properties such as NaOH, meglumine, and sorbitol are attached using a fluidized bed granulator. However, there were several problems in securing a stable monolayered formulation for telmisartan and amlodipine.

To solve this problem, we performed a salt screening which helped us identify that the potassium salt is ideal for achieving the highest stability. Telmisartan potassium used in our study is a non-hygroscopic substance. In addition, since the APIs (telmisartan and amlodipine) and insoluble alkalizer (MgO) used did not have deliquescent properties, the interaction between the two drugs did not occur, and impurities in the product were not detected during accelerated testing for 6 months.

During several sessions of discussion, qualitative PHA of CMAs(A) and CPPs(B) for initial risk assessment of single-layered TA tablets was established according to intended use and its type of dosage form for the preparation of coated TA tablets as shown in Table 2. The effect of CPPs and CMAs on CQAs must be defined early on in the process development of the pharmaceutical dosage form. Further risks associated with every step of the process, from the properties of the raw materials to the final product characteristics, were identified, analyzed, and evaluated thoroughly. The variables CMAs and CPPs affecting CQAs related to analytical methods, granule formulation, manufacturing process, and equipment performance were shown in the PHA (Table 2). As shown in Table 2A, the stabilizer and disintegrant were identified as qualitative risk CMAs. When the amount of the stabilizer, such as magnesium oxide, was increased, the inhibition of the dissolution rate could be predicted, and when the amount of the stabilizer was lower than optimum, the generation of the impurities could be rapidly increased. In addition, if the disintegrant deviated from the range of the optimum amount, there is a risk that the amount of the impurities may rapidly increase.

As shown in Table 2B, in the granulation process, speed and kneading time were identified as qualitative risk CPPs. If the kneading time was longer than the optimal condition, hard and large granules were formed, which could result in an out-of-similar dissolution rate of the reference drug or an increase in the impurities rate. Furthermore, in the tableting process, if the CPPs of turret speed and compression force were higher than optimum, the hardness and the disintegration of the tablets may be influenced, resulting in unevenness in the content, appearance, and dissolution rate of the tablets. As a result, in the initial RA, stabilizer and disintegrant were identified as the CMAs, and for the unit process, the granulation process and tablet compression were confirmed as CPPs.

### 3.2. Main Risk Assessment of the Critical Material Attributes and Critical Process Parameters Affecting Critical Quality Attributes

After selecting and evaluating the CMAs and CPPs that affect the CQAs by initial RA of qualitative PHA, quantitative failure mode and effect analysis (FMEA) was used for accuracy and precision analysis.

As shown in Table 2, the FMEA searching for CMAs was analyzed for failure mode and the effect on CQAs, and RPNs were calculated for each parameter by probability value (P), severity value (S), and detectability value (D). As shown in Table 1B, the FMEA searching for CPPs was included in the analysis, and the RPNs’ value was calculated for each parameter by P, S, and D. The values of P, S, and D for each failure mode in the case of the single-layered TA tablets were assigned based on previous experience and preliminary experiments, and the RPNs were calculated accordingly.

As shown in Table 2, the CMAs, the used stabilizer and disintegrant, were identified as the quantitative risk factors. It is indicated that a high RPN value for the non-optimal amount of stabilizer and disintegrant could generate a lag time in dissolution and disintegration. Furthermore, when the amount of the stabilizer is lower than optimum, the generation of the impurities may be rapidly increased. When the amount of the disintegrant is lower than optimum, the generation of the impurities may be rapidly increased. Thus, bioavailability and efficacy of the single-layered TA tablets may be compromised. So, the calculated RPN for this failure mode was a score of 48 when taking into consideration that the potential risk could be a non-optimal “disintegrant and stabilizer”.

As shown in Table 1B, the CPP of the kneading time of the wet granulation process was identified as the quantitative risk factor. It is indicated that a high RPN value for the non-optimal kneading time process could introduce an inadequate number of granules that could form agglomerates or lumps. Furthermore, when the kneading time was longer than optimum, appearance, assay, content uniformity, and dissolution profile of TA tablets may be affected. Thus, bioavailability and efficacy may be compromised. As a result, the calculated RPN for this failure mode was a score of 64 when taking into consideration that the potential risk could be an inadequate kneading time.

The CPPs of turret speed and compression force in the tablet compression were identified as the quantitative risk factors. It is indicated that a high RPN value for the non-optimal turret speed and compression force could introduce disordered tablets with inadequate dissolution, weight variation, and hardness or softness. Furthermore, when the turret speed and compression force is higher than optimum, the friability, appearance, weight variation, assay, disintegration time, dissolution rate, hardness, and content uniformity of the TA tablets may be affected. Thus, quality, safety, bioavailability, and efficacy may be compromised. Therefore, the calculated RPN for this failure mode was 48 when taking into consideration that the potential risk could be a non-optimal “turret speed and compression force”. As a result, in the main RA by FMEA, the risk factors that should be addressed in the DoE adopted by the RPN calculation were identified as stabilizer and disintegrant (CMAs) and kneading time, turret speed, and compression force as CPPs.

### 3.3. Experimental Design and Fitted Models

As shown in Table 3, two types of CMAs (stabilizer and a disintegrant) and one CPP (kneading time), through the PHA/FMEA of RA, were selected as three input (X) values for independent variables.

The experimental design was set in the range (three level) of 22–31–40 mg stabilizer, 1–2.8–4.6% disintegrant excipient, and 3.1–4.6–6.1 min of wet granulation (kneading) time. The output (Y) value was selected as the most important response to optimize the hardness, friability, disintegration, drug dissolution, and impurities selected as the dependent variables affected by the three X values. The experimental plan of 20 times runs (2^3^ + (2 × 3) + 6 = 20) was obtained and performed randomly.

As shown in Table 3, in the tablet compression process, a center composite design (CCD) of two factors with five levels was used to irradiate the effect of the independent variables on the dependent variables. The CPPs, compression force and turret speed, obtained through the PHA/FMEA of RA, were selected as the two input (X) values for independent variables. The experimental design was set in the range (five level) of 4.6–8–16–24–27.3 KN of compression force and 15.8–20–30–40–44.1 rpm of turret speed. Thus, the output (Y) value was selected as the most important response to optimize the hardness, friability, disintegration, drug dissolution, and content uniformity selected as the dependent variables affected by the two X values. A center composite design (CCD) was used for the DoE to optimize the CQAs according to the selected CPPs. The experimental plan of 13 times runs (2^2^ + (2 × 2) + 5) was obtained and performed randomly.

In the two processes, the mathematical response surface models were generated by statistical analysis using the Minitab 18^®^ software by applying coded values of factor levels. The software was also used for the plotting of the various three-dimensional (3D) plots, the analysis, and the design and contour plots.

For optimized modeling of the DoE, the response surface design analysis, through the ANOVA and the regression equation, was optimized by selecting only the significant factors. Finally, we confirmed whether the model was adequate through a lack of fit test. Since the *p*-value was >0.05, we could not refuse the null hypothesis for lack of conformity. After evaluating the main, interactive, and quadratic effects of factors related to the characteristics of single-layered TA tablets, we investigated the effect of each independent variable on the dependent variables. Variables with *p*-values <0.05 were regarded statistically significant. Therefore, the DoE modeling was optimized, and then the results were interpreted.

**Figure 2:**
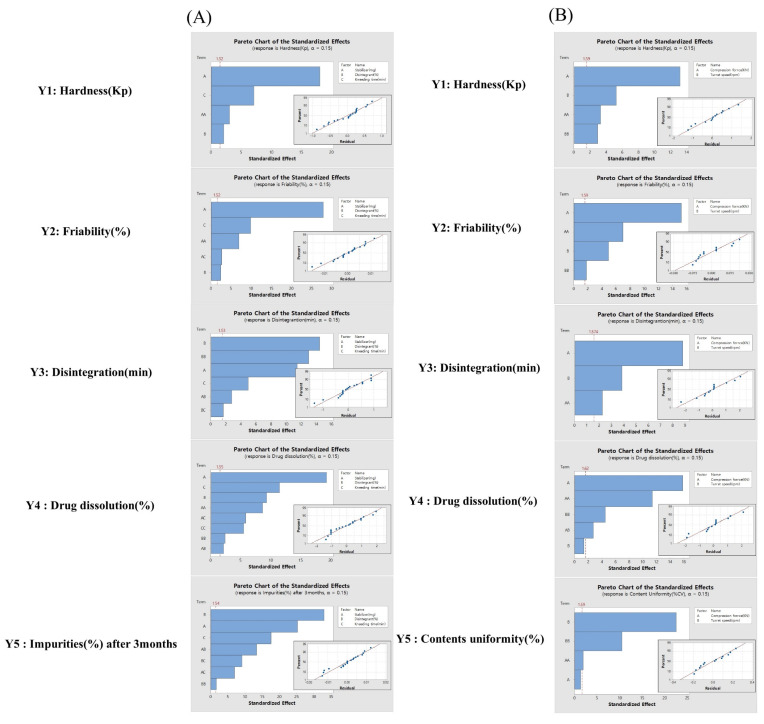
Effect analysis of CMAs and CPPs using a pareto chart and residual plot for wet granulation (**A**) and tablet compression (**B**).

**Figure 3:**
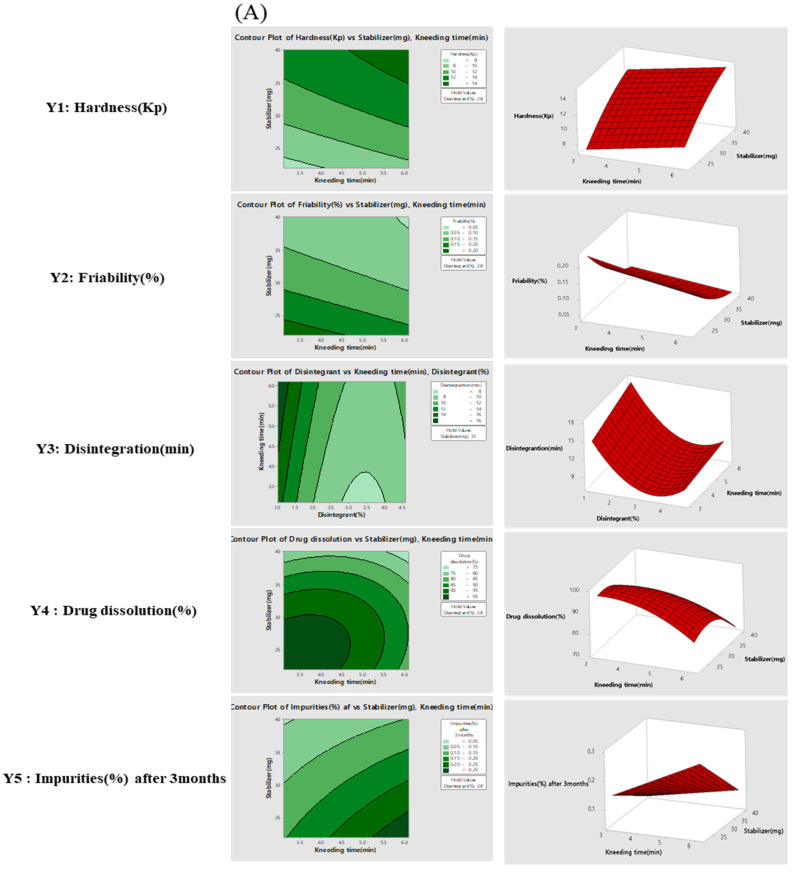

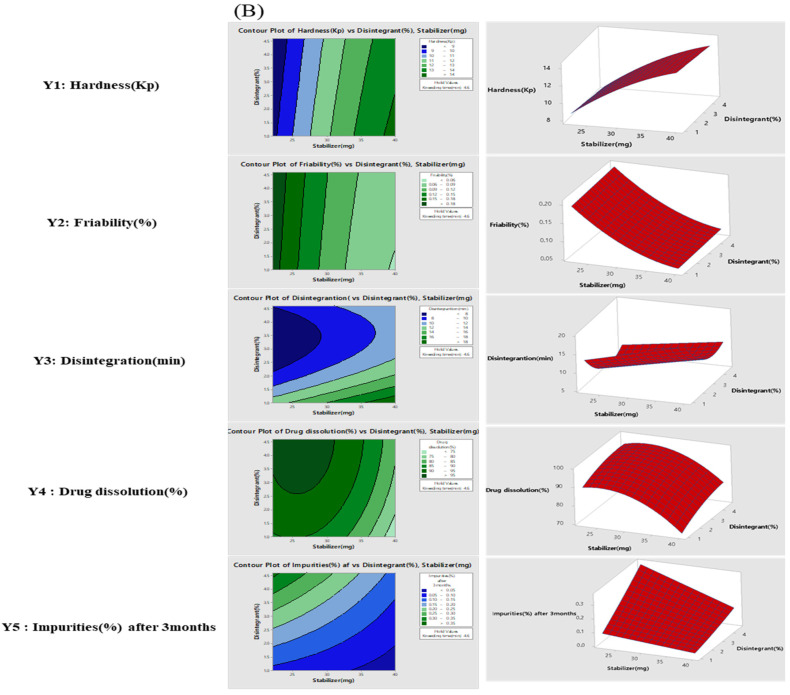
Effect analysis of CMAs and CPPs using contour plots and response surface plots for stabilizer and kneading time (**A**) and disintegrant and stabilizer (**B**) in the wet granulation.

As shown in Figure 2A and Figure 3A, the random behavior of the residuals was investigated with the residual analysis, and normal probability plots were confirmed for residual errors of the response variables. The residual plots showed straight lines and appeared to be normally distributed; outlier points were not observed in either one of the processes.

In the wet granulation process, the pareto chart was used to analyze the effect of the factors (Figure 2A). The independent variables that had the greatest effect on hardness and friability were identified as the stabilizer and kneading time. The independent variables that had the greatest effect on disintegration were identified as disintegrant. The independent variables that had the greatest effect on drug dissolution were identified as the stabilizer, kneading time, and the order of addition of disintegrant. The independent variables that had the greatest impact on impurities were the disintegrant type, stabilizer type, and kneading time sequences.

In the pareto chart of tablet compression process, the independent variable that had the greatest effect on hardness, friability, disintegration, and drug dissolution was identified as the compression force (Figure 2B). The independent variable that had the greatest effect on content uniformity was identified as the turret speed.

### 3.4. Development of Design Space

With the models developed and validated, the design space was finally optimized through a multidimensional combination of all individual acceptance regions for both CMA and CPP. The approach method of DoE led to an in-depth understanding of the influence of variables over the process.

In the wet granulation process, in the contour and response surface plots for hardness (Y1) and friability (Y2), we confirmed that with the increasing amount of stabilizer, the hardness increased. (Figure 3A), but the amount of disintegrant had little effect on hardness (Figure 3B). The contour and response surface plots of disintegration (Y3) showed that the disintegration time depended more on the specific stabilizer and concentration (%) of disintegrant than it did on the time of kneading time (Figure 3). The plots of drug dissolution (Y4) showed that the specific amount of stabilizer, kneading time, and disintegrant (%) sequences affected the dissolution values (Figure 3). The plots for total impurities of telmisartan and amlodipine (Y5) showed that the increase in impurities depended on the concentration of disintegrant and stabilizer, rather than on the kneading time.

**Figure 4:**
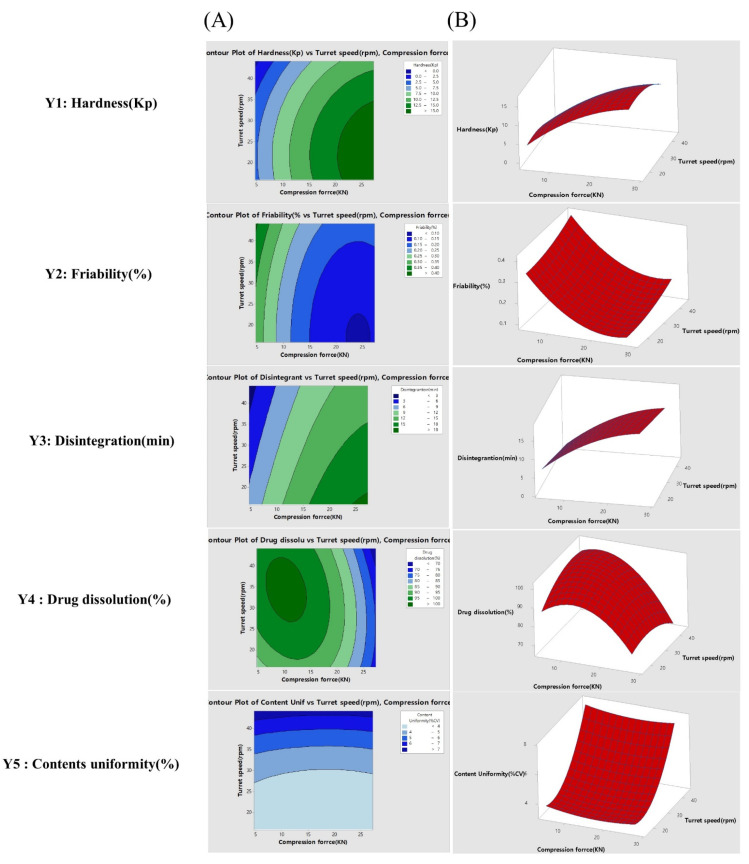
Effect analysis of CMAs and CPPs using contour plots (**A**) and response surface plots (**B**) for the tablet compression process.

In the tablet compression process, the contour and response surface plots for hardness (Y1), friability (Y2), and disintegration (Y3) showed that these factors depended more on the specific compression force (KN) than they did on the speed of turret (Figure 4). The plots of content uniformity (Y5) showed that these factors depended more on the specific the turret speed than they did on compression force (KN) (Figure 4). The plots for drug dissolution (%, Y5) showed that both compression force (KN) and turret speed (rpm) affected the dissolution values.

**Figure 5:**
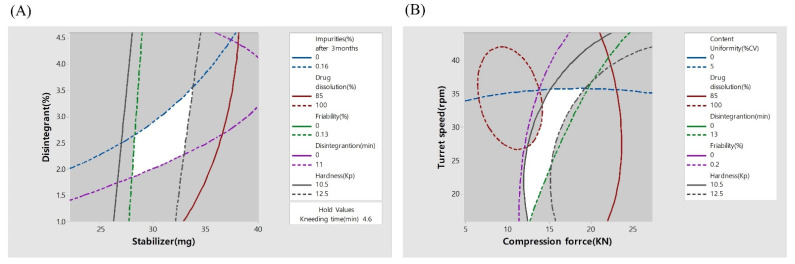
Design space of the wet granulations (**A**) and tablet compression process (**B**).

Figure 5 shows the optimum design space value of the actual response space by overlapping the contour plots shown in Figure 3 and Figure 4. By overlaying the contour plots from each response on top of each other, the design space was used to confirm the ideal window of operability space via the proven acceptable range and edges of failure, with relation to individual goals.

To set the design space of the two processes, the range of hardness in the contour plots was set to 10.5–12.5 Kp. The range of the friability was set up such that the maximum friability was not more than 0.13%. The range of the disintegration time was set up such that disintegration of the TA tablets was not more than 11 min. The range of drug dissolution was set such that the maximum drug dissolution after 30 min was not less than 85%. The range of total impurities was set such that the maximum impurities after 3 months was not more than 0.16%. The range of content uniformity (%) was set such that the maximum content uniformity was not more than 5.0%.

As mentioned above, the drug dissolution value after 30 min was the dissolution rate of telmisartan in the design space. Dissolution tests were performed for both telmisartan potassium and amlodipine besylate. However, amlodipine showed an immediate release pattern because of post-mixing, whereas telmisartan showed a dissolution delay pattern by various excipients. Therefore, the dissolution rate of telmisartan was considered to be the more critical factor.

Based on the area of the design space, it was possible to determine the space area that satisfied all the conditions. As a result, the single-layered TA tablets were prepared in the design space that satisfied all the conditions.

### 3.5. Pharmacokinetics

**Figure 6:**
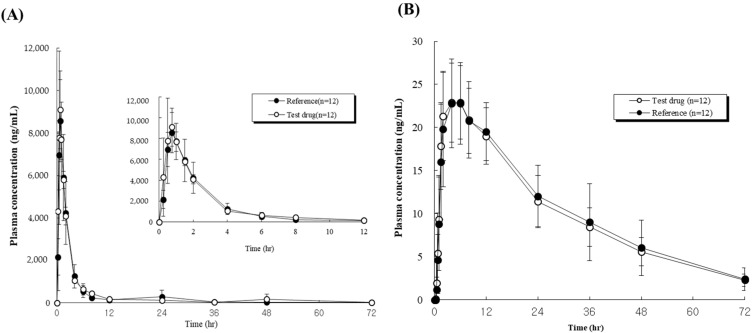
(**A**) Plasma concentration-time profiles of Twynstar^®^ and TA tablets at a dose of 80 mg telmisartan base in beagle dogs, and (**B**) Plasma concentration-time profiles of Twynstar^®^ and TA tablets at a dose of 5 mg amlodipine base in beagle dogs. Data represent the mean ± standard deviation (SD, n = 12).

The in vivo pharmacokinetic properties of the single-layered TA tablets, prepared by design space, after oral administration in beagle dogs (*n* = 12) were evaluated. The 80/5 mg of TA tablet concentrations were assessed from the plasma concentration-time profiles of Twynstar^®^ and the single-layered TA tablets at a dose of 8.0 mg/kg are indicated in Figure 6.

Figure 6A shows the plasma concentration-time profiles of Twynstar^®^ and TA tablets at a dose of 80 mg telmisartan base. Figure 6B shows the plasma concentration-time profiles of Twynstar^®^ and TA tablets at a dose of 5 mg amlodipine base.

As shown in Table 4, in the case of the bioequivalence of telmisartan administration, after several oral administrations to beagles, the AUC of the Twynstar^®^ and TA tablets (25,527.5 ± 3808.5 and 27,386.2 ± 5269.5 ng·h^−1^·mL^−1^, respectively) were not significantly different (*p* > 0.05), which indicated that they had similar circulation times and similar absorption into the blood. The 90% confidence interval (CI, 0.8963–1.0952) of the geometric least square mean ratios of the AUC was 0.9321 of the point estimate, which was within 0.80–1.25 of the bioequivalence criteria for telmisartan.

The C_max_ values of the Twynstar^®^ and TA tablets (8925.2 ± 1474.7 and 9486.4 ± 1495.3 ng/mL, respectively) were not considerably different (*p* > 0.05), which indicated that they had similar absorption rates. Furthermore, the 90% CI (0.9004–1.0895) of the C_max_ was 0.9408 of the point estimate, which was within the 0.80–1.25 range of the bioequivalence criteria of telmisartan. These results indicate that the Twynstar^®^ and TA tablets for telmisartan have similar absorption rates following oral administration. The 90% CI of the geometric mean ratio of Cmax (test/reference drug) indicates that it is within the acceptable range in the in vivo experiment in beagles, which is shown in Figure 6B.

As shown in Table 5, in the case of the bioequivalence of amlodipine administration, after several oral administrations to beagle dogs, the AUC of the Twynstar^®^ and TA tablets (737.7 ± 198.04 and 712.9 ± 154.65 ng·h^−1^·mL^−1^, respectively) were not significantly different (*p* > 0.05), which indicated that they had similar absorption and circulation times. A total of 90% CI (0.9334–1.0226) of the geometric least square mean ratios of the AUC was 0.9770 of the point estimate, which was within 0.80–1.25 of the bioequivalence criteria for amlodipine besylate. The C_max_ values of the Twynstar^®^ and TA tablets (23.88 ± 4.58 and 23.55 ± 5.06 ng/mL, respectively) were not significantly different (*p* > 0.05), which indicated that they had similar absorption rates. Furthermore, 90% CI (0.9163–1.0510) of the C_max_ was 0.9813 of the point estimate, which was within the 0.80–1.25 range of the bioequivalence criteria of amlodipine. These results indicate that the Twynstar^®^ and TA tablets for amlodipine have similar absorption rates following oral administration for bioequivalences. A total of 90% CI of the geometric mean ratio of Cmax (test/reference drug) indicates that it is within the acceptable range in the in vivo experiment in beagles, which is shown in Figure 6B.

Therefore, this preclinical study demonstrated that the test and reference product were bioequivalent, indicating that the monolayered TA tablets could be an appropriate alternative for the commercial product (Twynstar^®^).

## 4. Conclusions

Our research team developed a novel single-layered TA tablet, containing telmisartan and amlodipine, by applying the QbD approach to the manufacturing of pharmaceutical products. Consequently, we achieved a higher product yield (95–100%) of single-layered tablets than that of the conventional double-layered tablet (90–95%). The developed tablet maintained the same efficacy and bioequivalence as its double-layered commercially-available counterpart, Twynstar^®^, and solved the problems relating to the quality, impurities, and manufacturing methods, without using a double-layer tablet press. This confirmed that, irrespective of the manufacturing costs, there are possible high quality improvements for manufacturing processes. The size of the tablet we developed was reduced by 50% or more when compared to Twynstar^®^, thereby improving the ease of swallowing, especially in elderly patients with hypertension.

Thus, the single-layered TA tablet could be a promising candidate as a possible alternative for patients who require a telmisartan/amlodipine combination. For the further development of the TA tablet, a study on its bioequivalence in human subjects is recommended.

## Figures and Tables

**Table 1:** Main risk assessment of the critical material attributes (A) and critical process parameters (B) affecting critical quality attributes.

(A)							
**Functions**	**MAs (No color)** **CMAs (Color)**	**Failure Mode**	**Effect on CQAs as justification of failure mode**	**P**	**S**	**D**	**RPN**
**Telmisartan potassium**	**Salt form/PSD/solubility/dissolution**	**polymorphism/Higher PSD**	**Drug solubility and dissolution may be affected. Thus, bioavailability and efficacy may be compromised.**	**3**	**4**	**1**	**12**
**Amlodipine besylate**	**PSD/solubility/dissolution**	**Higher PSD**	**Drug solubility and dissolution may be affected. Thus, bioavailability and efficacy may be compromised.**	**3**	**4**	**1**	**12**
**Stabilizer**	**Amount of stabilizer**	**Higher than optimum**	**While maintaining high stability of tablet, produces hard granules yielding tablets with longer disintegration and dissolution times. Thus, bioavailability and efficacy may be compromised.**	**4**	**4**	**3**	**48**
		**Lower than optimum**	**While maintaining low stability of tablet, formation of loose and soft granules may produce friable tablets that dissolve immediately. Thus, bioavailability and efficacy may be compromised.**	**4**	**4**	**3**	**48**
**Diluent**	**PSD**	**Uneven**	**Flow properties of the blend and content uniformity may be affected in dry mixing process. Thus, quality/safety may be compromised.**	**3**	**3**	**2**	**18**
	**Moisture content**	**High**	**Impurity profile may be affected. Thus, safety may be compromised.**	**3**	**3**	**2**	**18**
**Binding solution**	**Volume of binding solution**	**Higher than optimum**	**Produces hard granules yielding tablets with longer disintegration and dissolution times. Thus, bioavailability and efficacy may be compromised.**	**4**	**4**	**2**	**32**
		**Lower than optimum**	**Formation of loose and soft granules may produce friable tablets that dissolve immediately. Thus, bioavailability and efficacy may be compromised.**	**4**	**4**	**2**	**32**
**Disintegrant**	**Amount of disintegrant agent**	**Higher than optimum**	**Desired dissolution of reference tablet could not be achieved because it showed a high dissolution pattern. Thus, bioavailability and efficacy may be compromised.**	**4**	**4**	**3**	**48**
		**Lower than optimum**	**Desired dissolution of reference tablet could not be achieved because it showed a high dissolution pattern. Thus, bioavailability and efficacy may be compromised.**	**4**	**4**	**3**	**48**
**Glidant**	**Concentration of glidant**	**Lower than optimum**	**Flow of granules/powder from hopper to die may be affected due to reduced friction between particles. Therefore, content uniformity and product quality may be compromised.**	**3**	**3**	**2**	**18**
**Lubricant**	**Concentration of lubricant**	**Lower than optimum**	**Tablet granules/powder may stick to the surfaces of punches/tools, and picking may be observed. Thus, product quality may be compromised.**	**3**	**3**	**2**	**18**
		**Higher than optimum**	**Hydrophobic lubricant may coat drug particle surfaces, thereby retarding dissolution. Thus, efficacy may be compromised.**	**3**	**3**	**3**	**27**
(B)							
**Unit processing**	**PPs (No color)** **CPPs (Color)**	**Failure Mode**	**Effect on CQAs as justification of failure mode**	**P**	**S**	**D**	**RPN**
**Screening**	**Delumping or Sifting**	**Larger than optimum sieve size**	**Un-plain particle size distribution could cause content non-uniformity. Weight variation, assay, and content uniformity may be affected. Thus, safety and quality may be compromised.**	**2**	**3**	**2**	**12**
**Blending**	**Speed and time of dry mixing**	**Lower mixing speed and shorter time**	**Weight variation, assay, and content uniformity may be affected. Thus, safety and quality may be compromised.**	**2**	**3**	**2**	**12**
**Granulation**	**Impeller speed and time**	**Higher mixing speed and longer time**	**Produces large granules that form agglomerates or lumps. Appearance, assay, content uniformity, and dissolution profile of tablets may be affected. Bioavailability and efficacy may be compromised.**	**4**	**4**	**2**	**32**
	**Chopper speed and time**	**Lower mixing speed and shorter time**	**Produces large granules that form agglomerates or lumps. Appearance, assay, content uniformity, and dissolution profile of tablets may be affected. Bioavailability and efficacy may be compromised.**	**4**	**4**	**2**	**32**
	**Kneading time for granulation**	**Longer than optimum time**	**Produces large granules that form agglomerates or lumps. Appearance, assay, content uniformity, and dissolution profile of tablets may be affected. Bioavailability and efficacy may be compromised.**	**4**	**4**	**4**	**64**
**Drying**	**Inlet temperature**	**Lower than optimum temperature**	**Physical appearance and powder quality may be affected. Both sticking and picking may be observed. Thus, product quality and tablet compression may be compromised.**	**3**	**3**	**2**	**18**
		**Higher product temperature**	**Degradation and impurity profile may be affected. Thus, safety may be comprised.**	**3**	**4**	**2**	**24**
**Blending and lubrication**	**Blending speed and time**	**Higher than optimum speed and longer time**	**Dissolution time may be increase. Thus, efficacy may be compromised.**	**3**	**2**	**2**	**12**
**Tablet Compression**	**Speed of turret and feeder**	**Higher than optimum speed**	**Appearance, weight variation, assay, and content uniformity may be affected. Thus, quality, safety, and efficacy may be compromised.**	**4**	**4**	**3**	**48**
	**Compression force** **(Pre-compression and main compression force)**	**Higher than optimum force**	**Friability, appearance, disintegration, dissolution, and hardness of tablets may be affected. Thus, quality, bioavailability and efficacy may be compromised.**	**4**	**4**	**3**	**48**
**Film coating**	**Product temperature**	**Higher than optimum temperature**	**Impurity level and degradation profile may be affected. Thus, appearance of product, stability and safety may be compromised.**	**3**	**3**	**3**	**27**

**Table 2:** Initial risk assessment of the critical material attributes (A) and critical process parameters (B) affecting critical quality attributes.

(A)								
**CMAs affecting CQAs**	**Telmisartan potassium**	**Amlodipine besylate**	**Stabilizer**	**Diluent**	**Binding solution**	**Disintegrant**	**Glidant**	**Lubricant**
**Appearance**	**Low**	**Low**	**Low**	**Low**	**Low**	**Medium**	**Low**	**Medium**
**Assay**	**Low**	**Low**	**Low**	**Low**	**Low**	**Low**	**Low**	**Low**
**Uniformity**	**Low**	**Low**	**Low**	**Medium**	**Low**	**Low**	**Low**	**Low**
**Impurities**	**Medium**	**Medium**	**High**	**Low**	**Low**	**High**	**Low**	**Low**
**Dissolution**	**Medium**	**Low**	**High**	**Low**	**Medium**	**High**	**Medium**	**Medium**
(B)								
**CPPs affecting CQAs**	**Screening**	**Blending**		**Granulation**	**Drying**	**Blending and lubrication**	**Tablet compression**	**Film coating**
**Appearance**	**Low**	**Low**		**Medium**	**Low**	**Low**	**Medium**	**Low**
**Assay**	**Medium**	**Low**		**Medium**	**Low**	**Low**	**Medium**	**Low**
**Uniformity**	**Medium**	**Medium**		**Medium**	**Low**	**Low**	**Medium**	**Low**
**Impurities**	**Low**	**Low**		**High**	**Medium**	**Low**	**Low**	**Medium**
**Dissolution**	**Low**	**Low**		**High**	**Low**	**Medium**	**High**	**Low**

**Table 3:** Experimental matrix and measured responses values for optimization of the TA tablet using face centered central composite design for wet granulation (A), and central composite design for tablet compression (B).

(A)								
	Critical material attributes		Critical process parameters	Critical quality attributes				
Run	X1	X2	X3	Y1	Y2	Y3	Y4	Y5
	Stabilizer (mg)	Disintegrant (%)	Kneading time (min)	Hardness (Kp)	Friability (%)	Disintegration (min)	Drug dissolution (%)	Impurities (%) after 3 months
1	22	1	3.1	7.6	0.230	12.2	93 ± 3.4	0.052
2	40	1	3.1	13.4	0.077	17.8	70 ± 5.2	0.017
3	22	4.6	3.1	7.5	0.230	7.8	97 ± 1.9	0.249
4	40	4.6	3.1	12.2	0.086	11.1	78 ± 2.9	0.081
5	22	1	6.1	8.9	0.163	14.4	77 ± 5.0	0.123
6	40	1	6.1	15.4	0.048	21.1	65 ± 4.7	0.037
7	22	4.6	6.1	9.4	0.173	8.9	83 ± 3.8	0.475
8	40	4.6	6.1	14.9	0.048	12.2	75 ± 4.4	0.178
9	22	2.8	4.6	8.4	0.202	5.6	96 ± 1.2	0.220
10	40	2.8	4.6	13.8	0.058	12.2	77 ± 2.4	0.077
11	31	1	4.6	12.9	0.086	16.7	86 ± 3.2	0.062
12	31	4.6	4.6	10.8	0.125	10.0	97 ± 1.3	0.261
13	31	2.8	3.1	10.2	0.134	7.8	94 ± 3.2	0.099
14	31	2.8	6.1	13.2	0.086	11.1	84 ± 4.2	0.195
15	31	2.8	4.6	12.4	0.106	8.9	92 ± 1.3	0.149
16	31	2.8	4.6	12.1	0.106	7.8	92 ± 3.2	0.149
17	31	2.8	4.6	11.3	0.115	8.9	94 ± 2.5	0.145
18	31	2.8	4.6	12.1	0.106	8.9	93 ± 2.6	0.147
19	31	2.8	4.6	11.4	0.115	10.0	92 ± 1.3	0.147
20	31	2.8	4.6	11.9	0.096	10.0	94 ± 2.0	0.146
(B)								
	Critical process parameters		Critical quality attributes					
Run	X1	X2	Y1	Y2	Y3		Y4	Y5
	Compression force (KN)	Turret speed (rpm)	Hardness (Kp)	Friability (%)	Disintegration (min)		Drug dissolution (%)	Content Uniformity (%CV)
1	8	20	7.5	0.25	11		94.5 ± 1.4	3.51
2	24	20	15	0.12	16		82.5 ± 3.0	3.56
3	8	40	5.4	0.3	7		98.5 ± 1.1	6.46
4	24	40	13.2	0.14	13.7		78.5 ± 5.2	6.20
5	4.686	30	3	0.37	2		98.5 ± 1.4	4.27
6	27.314	30	16.5	0.11	17		75.5 ± 4.4	3.95
7	16	15.858	13.5	0.13	15		92.5 ± 1.0	3.28
8	16	44.142	6.53	0.23	9.3		96.5 ± 0.9	7.21
9	16	30	12.2	0.16	12.2		98.5 ± 1.2	4.01
10	16	30	12.3	0.16	11.8		98.5 ± 1.3	4.14
11	16	30	12.5	0.15	12.5		98.2 ± 1.5	3.79
12	16	30	12.4	0.15	12.5		97.9 ± 1.0	3.89
13	16	30	11.9	0.16	12.1		98.5 ± 1.2	4.08

**Table 4:** Pharmacokinetic parameters of Twynstar^®^ and TA tablets at a dose of 80 mg telmisartan base in beagle dogs.

Telmisartan	Reference Drug ^1^	Test Drug ^2^	Point Estimate	90% CI ^3^
	Mean ± SD			
Tmax ^4^ (h)	0.67 ± 0.14	0.67 ± 0.14	-	-
Cmax ^5^ (ng/mL)	8925.2 ± 1474.7	9486.4 ± 1495.3	0.9408	0.9004–1.0895
AUC_(last)_ ^6^ (ng∙h^−1^∙mL^−1^)	25,527.5 ± 3808.5	27,386.2 ± 5269.5	0.9321	0.8963–1.0952
AUC_(inf)_ ^6^ (ng∙h^−1^∙mL^−1^)	26,021.7 ± 3513.6	28,263.1 ± 5933.2	0.9206	-
T_1/2_^7^(h)	19.6 ± 14.5	24.5 ± 24.2	-	-

^1^ Reference drug: Twynstar^®^ (Telmisartan + Amlodipine) double-layered tablets. ^2^ Test drug: monolayered telmisartan potassium and amlodipine besylate (TA) tablets. ^3^ 90% CI: 90% confidence interval. ^4^ T_max_: time to C_max_. ^5^ C_max_: maximum plasma concentration. ^6^ AUC: area under the plasma concentration-time curve. ^7^ T_1/2_: half-life.

**Table 5:** Pharmacokinetic parameters of Twynstar^®^ and TA tablets at a dose of 5 mg amlodipine base in beagle dogs.

Amlodipine	Reference Drug ^1^	Test Drug ^2^	Point Estimate	90% CI ^3^
	Mean ± SD			
Tmax ^4^ (h)	4.83 ± 1.34	4.33 ± 1.44	-	-
Cmax ^5^ (ng/mL)	23.88 ± 4.58	23.55 ± 5.06	0.9813	0.9163–1.0510
AUC_(last)_ ^6^ (ng∙h^−1^∙mL^−1^)	737.7 ± 198.04	712.9 ± 154.65	0.9770	0.9334–1.0226
AUC_(inf)_ ^6^ (ng∙h^−1^∙mL^−1^)	807.3 ± 225.72	778.6 ± 170.03	0.9644	-
T_1/2_^7^(h)	19.5 ± 3.94	19.5 ± 3.45	-	-

^1^ Reference drug: Twynstar^®^ (Telmisartan + Amlodipine) double-layered tablets. ^2^ Test drug: monolayered telmisartan potassium and amlodipine besylate (TA) tablets. ^3^ 90% CI: 90% confidence interval. ^4^ T_max_: time to C_max_. ^5^ C_max_: maximum plasma concentration. ^6^ AUC: area under the plasma concentration-time curve. ^7^ T_1/2_: half-life.

## Data Availability

The data presented in this study are included in this published article.
